# Age Matters: The Impact of Ageism on Health, Cognitive Impairment, Work Lives, and Anti-Ageist Interventions

**DOI:** 10.1093/geroni/igaa057.2329

**Published:** 2020-12-16

**Authors:** Philip Rozario, Nancy Morrow-Howell

## Abstract

Age is a social constructions. The treatment of people on the basis of their age, imposes serious psycho-emotional, social and economic costs on society and older people. The experiences of ageism may be exacerbated when other forms of acute and chronic forms of oppression are experienced due to racism, sexism, etc. The first paper looks at the impact of ageism on older people’s health. Their systematic review of studies showed that ageism detrimentally and consistently impacted older individuals in 11 health domains, with the prevalence of significant findings increasing over time. Informed by NIA’s Health Disparities Framework, the second paper examines the relationships between discrimination and protective factors on cognitive functioning. Their analyses of the Health and Retirement Survey data reveal, among other things, that everyday experiences of ageism significantly worsen older adults cognitive functioning. Using survey data of adults throughout the life-span, our third presenter examines how multiple identities (such as age and gender) influence employees’ perspectives on workplace fairness in hypothetical situations. The findings are informative to human resource departments in ensuring workplace fairness within the context of a multigenerational workforce. The final paper examines two efforts to disrupt ageism, specifically interventions that target students in an intergenerational program and staff members of senior living communities. Results from these evaluations point to an increase in positive attitudes among students and a reduction in ageist behaviors among staff members. All presenters will discuss policy, practice and research implications of their studies.

